# Traumatic Stress Produces Delayed Alterations of Synaptic Plasticity in Basolateral Amygdala

**DOI:** 10.3389/fpsyg.2019.02394

**Published:** 2019-10-25

**Authors:** Huan-Huan Zhang, Shi-Qiu Meng, Xin-Yi Guo, Jing-Liang Zhang, Wen Zhang, Ya-Yun Chen, Lin Lu, Jian-Li Yang, Yan-Xue Xue

**Affiliations:** ^1^Department of Psychiatry, Tianjin Medical University, Tianjin, China; ^2^Department of Clinical Psychology, Tianjin Medical University General Hospital, Tianjin, China; ^3^National Institute on Drug Dependence, Peking University, Beijing, China; ^4^State Key Laboratory of Natural and Biomimetic Drugs, Department of Molecular and Cellular Pharmacology, Peking University School of Pharmaceutical Sciences, Beijing, China; ^5^Department of Medicinal Chemistry and Molecular Pharmacology, Purdue University College of Pharmacy and Purdue Institute for Integrative Neuroscience, West Lafayette, IN, United States; ^6^Peking University Sixth Hospital/Peking University Institute of Mental Health, Peking University, Beijing, China

**Keywords:** single prolonged stress, post-traumatic stress disorder, dendritic spines, synaptic plasticity, basolateral amygdala

## Abstract

Acute traumatic event exposure is a direct cause of post-traumatic stress disorder (PTSD). Amygdala is suggested to be associated with the development of PTSD. In our previous findings, different activation patterns of GABAergic neurons and glutamatergic neurons in early or late stages after stress were found. However, the neural plastic mechanism underlying the role of basolateral amygdala (BLA) in post-traumatic stress disorder remains unclear. Therefore, this study mainly aimed at investigating time-dependent morphologic and electrophysiological changes in BLA during the development of PTSD. We used single prolonged stress (SPS) procedure to establish PTSD model of rats. The rats showed no alterations in anxiety behavior as well as in dendritic spine density or synaptic transmission in BLA 1 day after SPS. However, 10 days after SPS, rats showed enhancement of anxiety behavior, and spine density and frequency of miniature excitatory and inhibitory postsynaptic currents in BLA. Our results suggested that after traumatic stress, BLA displayed delayed increase in both spinogenesis and synaptic transmission, which seemed to facilitate the development of PTSD.

## Introduction

As an intricate anxiety disorder, post-traumatic stress disorder (PTSD) generally occurs after traumatic stress exposure (Galea et al., 2007; Keyes et al., 2013; Scott et al., 2013; Olaya et al., 2015). PTSD has a high prevalence rate worldwide (Seal et al., 2009) and imposes a heavy burden to families and the society (Cohen et al., 2010). But the biological basis underlying PTSD was unclear. Single prolonged stress (SPS) model, an appropriate PTSD model of animal, has been established to explore the neurobiological mechanisms of PTSD considering the limitations of human studies (Souza et al., 2017; Fang et al., 2018). Rats exhibited abnormal behavior as well as hypothalamic-pituitary-adrenal (HPA)-axis dysfunction following SPS (Ding et al., 2010), which is a putative neuroendocrinological hallmark of PTSD (Mellman et al., 2009; Hughes and Shin, 2011; Pratchett et al., 2011; Bailey et al., 2013). SPS paradigm is composed of the following procedures (Bradley et al., 2005): restraint, forced swim in water at 20–24°C, ether exposure, and stay at homecage undisturbedly for 7 days which is essential for the development of key symptoms of PTSD (Liberzon et al., 1997; Knox et al., 2012b). This model can mimic the symptoms of PTSD in humans, with behavioral changes including increased anxiety (Han et al., 2014; Fang et al., 2018), impaired social interaction and spatial memory (Wen et al., 2016), and disrupted extinction of fear memory (Iwamoto et al., 2007; Fang et al., 2018).

The amygdala, which is involved in the regulation of fear and memory (Dias et al., 2014) and emotion (Saghir et al., 2018; Abuhasan and Siddiqui, 2019), is located at the limbic system of the brain and consists of several subregions, such as corticomedial nucleus, basolateral nucleus (BLA), central nucleus. Pyramidal neurons account for about 85% of all neurons in the adult BLA, and the rest are mainly interneurons (Berdel et al., 1997; Duvarci and Pare, 2014). It has been suggested that the dysfunction of amygdala is associated with the pathogenesis of mental disorders, such as depression, anxiety, and autism (Rainnie et al., 2004; Shekhar et al., 2005; Truitt et al., 2007; Koob and Volkow, 2010). The clinical study on PTSD has revealed that the response of amygdala in patients to emotional stimuli was exaggerated (Rauch et al., 2000). Furthermore, amygdala’s response to fear stimuli could be used to evaluate the treatment effect (Bryant et al., 2008). A series of molecular substrates in amygdala have been implicated in the PTSD-associated behaviors, such as glucocorticoid receptor (Kohda et al., 2007; Cohen et al., 2012), betaarrestin-2 (Ding et al., 2017), β-adrenoreceptor (Ronzoni et al., 2016), and mTOR signaling pathway (Oh et al., 2018). We also recently found different activating patterns of glutamatergic and GABAergic neurons in amygdala, specifically delayed enhancement of glutamatergic pyramidal neuron activation in BLA (Fang et al., 2018). However, fewer studies have showed how the synaptic plasticity in BLA changes in the animal model of PTSD (Cohen et al., 2014).

Dendrites and dendritic spines form the structural basis of synaptic plasticity (Spruston, 2008; Papoutsi et al., 2014). Neural circuits are shaped with dendritic morphology, and generation and storage of memory involves adjustment of structures of spines and dendrites in the brain (Papoutsi et al., 2014). Dendrites in amygdala are especially sensitive to stress. Significant changes have been exhibited in spine density of pyramidal neurons as well as dendritic morphology in amygdala in rats that underwent chronic or acute stress (Vyas et al., 2002, 2006; Mitra et al., 2005; Leuner and Shors, 2013; Padival et al., 2013; Suvrathan et al., 2014; Yasmin et al., 2016). Dendritic spines are usually classified according to shapes (stubby, thin, mushroom) (Wang et al., 2017), which are distinct in their functions (Noguchi et al., 2005; Bourne and Harris, 2007; Gipson and Olive, 2017). Studies have shown that the number of postsynaptic glutamatergic receptors decides the spine morphology to a great extent (Rochefort and Konnerth, 2012). Taken together, in order to identify changes in synaptic plasticity in BLA after traumatic stress, we used Golgi–Cox method to determine if SPS causes alterations of spine morphology and density, and recorded mEPSCs and mIPSCs to explore whether SPS leads to activity alterations in excitatory synapses and inhibitory synapses.

## Materials and Methods

### Subjects

Sprague-Dawley rats (3-month old, male), with weight of 220–260 g, were gained from the Laboratory Animal Center, Peking University Health Science Center. The rats were kept in groups of five at temperature of 23 ± 2°C and humidity of 50 ± 5% with free access to water and food under a 12 h:12 h light:dark cycle. We performed all the behavioral experiments under the dark phase. Animal care and experimental procedures were conducted according to the National Institutes of Health Guide for the Care and Use of Laboratory Animals. All experiments were permitted by Biomedical Ethics Committee of Peking University.

### Single Prolonged Stress

Single prolonged stress is a commonly recognized PTSD model (Ding et al., 2010), which results in potent responses to stress via psychological (restraint), physiological (forced swimming), and pharmacological (exposure to ether) pathways. The SPS procedure was conducted based on previous study (Fang et al., 2018), including 2-h restraint, 20-min forced swimming, recovery in homecage for 15 min, and exposure to diethyl ether until a brief loss of consciousness. On the same day of SPS treatment, the control animals were handled. All animals were undisturbed in their homecages for 10 days before sensitization test (Liberzon et al., 1997).

### Open Field Test

The apparatus of the open field test (OFT) had a square arena at 75 × 75 × 40 cm and was divided into 25 even squares with size of 15 × 15 cm (Xue et al., 2015; Fang et al., 2018). The apparatus was illuminated at 10 lux. Towels soaked with 75% ethanol were used to clear up the apparatus and wipe off odor of previous rat after each 5-min run. A rat was put in the center of the apparatus, and its movement was recorded by a digital video camera mounted on the roof and connected to a computer. Using an EthoVision System XT 10.1 (Noldus Information Technology, Netherlands), the time spent in the central part of the apparatus was analyzed.

### Elevated Plus Maze

The elevated plus maze (EPM) test was conducted as previously described (Xue et al., 2015; Fang et al., 2018). Two open arms (50 × 10 cm), and two closed arms (50 × 10 × 40 cm), as well as a middle compartment (10 × 10 cm) constituted the shape of a plus, which were placed 70 cm above the ground. Each rat explored the apparatus *ad libitum* for 5 min after being placed in the middle compartment with head facing an open arm. Towels soaked with 75% ethanol were used to clear up the apparatus and wipe off odor of the previous rat after each run. Movement of rats was recorded using a video camera mounted on the roof and connected to a computer. The test was performed with illuminance level of 3 lx in the closed arms and 8 lx in the open arms (Suo et al., 2013). Using an EthoVision System XT 10.1 (Noldus Information Technology, Netherlands), the number of entries into the open arms and time spent (sec) in the open arms were analyzed.

### Slice Preparation

The brains were rapidly removed after rats were anesthetized and then decapitated. The brains were immediately placed into cutting solution (in mM) at 0–4°C: 87 NaCl, 3.0 KCl, 1.5 CaCl_2_, 1.3 MgCl_2_, 1.0 NaH_2_PO_4_, 26 NaHCO_3_, 20 D-glucose, and 75 sucrose, saturated with 95% O_2_ and 5% CO_2_ to obtain 250 μm-thick coronal sections with a vibratome (Leica VT1000 S). Transverse slices containing the BLA were cut and transferred into a holding chamber containing ACSF (in mM): 124 NaCl, 3.0 KCl, 1.5 CaCl_2_, 1.3 MgCl_2_, 1.0 NaH_2_PO_4_, 26 NaHCO_3_, and 20 D-glucose, saturated with 95% O_2_ and 5% CO_2_ at 33°C for 30 min and then at room temperature for at least 30 min until being used for recordings.

### Whole-Cell Patch-Clamp Recording

Neurons with obvious primary dendrites and spines were selected, which is the morphological characteristics of BLA principal neurons (McDonald, 1982; Padival et al., 2013). Whole-cell patch clamp pipettes were composed of borosilicate glass capillaries (1.5 mm outer diameter; World Precision Instruments, Sarasota, FL, United States). The resistances of electrodes were from 2 to 3.5 MΩ. Voltages were corrected for a liquid junction potential of 13–14 mV, calculated using pClamp 10.3. Recordings were performed at 32–33°C, with stable perfusion of ACSF (2 ml/min). Electrodes were filled with (in mM): 110 Cs methylsulfate, 0.3 Tris-GTP, 15 CsCl, 2 MgCl_2_, 0.5 EGTA, 10 HEPES, 4 ATP-Mg, 4 QX-314 and 5 Na_2_-phosphocreatine (pH 7.15–7.25 with CsOH, 270–280 mOsm with sucrose). To record miniature synaptic events (mEPSCs and mIPSCs), we bathed the slices in normal ACSF containing 1.0 μM TTX. After allowing for 5 min of stabilization after break in, mEPSCs and mIPSCs were recorded at a holding potential of −70 mV for 2 min and 0 mV for 2 min, respectively (Lippi et al., 2016; Nagode et al., 2017). The postsynaptic currents recorded at −70 mV were blocked after the addition of 20 μM CNQX and 50 μM AP5, whereas those recorded at 0 mV were blocked by 50 μM picrotoxin (Supplementary Figure S1). Series resistance was constantly monitored. Cell input resistance (R_*in*_) was calculated by determining the current response from a holding potential of −70 mV to the steps of −5 mV hyperpolarization (Mizunuma et al., 2014; Nagode et al., 2017). Data were excluded when the series resistance reached above 16 MΩ or the change of series resistance reached more than 20%. In this study, the rise time represents 10–90% rise time, and the decay kinetics were measured as 90–37% decay time. The total number of events that occurred during 2-min recording epochs was analyzed. The number of mEPSCs used in the analysis for each cell ranged from 109 to 942, and the number of mIPSCs ranged from 55 to 899. Previous study showed that the mEPSC rise time was variable depending on the different electrotonic distances from the somatic recording site to the synaptic region where each mEPSC occurs, and events originating from the soma or dendrites presented as fast and slow rising events respectively (Han et al., 2013). In the present study, we did not detect the specific populations of mEPSCs via the rising time, and no criteria relating to rise time were used to further filter detected events. Thus, the mEPSCs and mIPSCs may be comprised of both proximal (i.e., somatic) and distal (dendritic) events. In this study, mEPSCs and mIPSCs were analyzed by the two exponential equations model fitting in Decay fit of MiniAnalysis software. This method was fit to an ensemble average generated for each cell. The equation is as follows: y = A1^∗^e^(-x/t1) + A2^∗^e^(-x/t2). Signals were amplified with a MultiClamp 700B amplifier (Molecular Devices, Union City, CA, United States), filtered at 2 kHz, and digitized at 10 kHz. Data were analyzed with the pCLAMP 10.3 data acquisition program (Molecular Devices). Miniature events were detected offline using MiniAnalysis (Synaptosoft), with the amplitude threshold set to 5 pA and an area threshold of 10.

### Golgi-Cox Staining

Rats were anesthetized and transcardially perfused with 0.9% normal saline solution. Brains were dissected, and were immersed with a Golgi-Cox solution for 2 weeks based on previous studies (Yang et al., 2015; Han et al., 2016; Wang et al., 2017), and then in 30% sucrose solution for 2–5 days in darkness at room temperature. Coronal sections (200 μm) were prepared using a vibratome (Microm HM 650V, Thermo Scientific, Walldorf, Germany) according to previous studies (Yang et al., 2015; Wang et al., 2017). Slides were kept in the darkness during staining and afterward.

Neurons with obvious primary dendrites and spines were selected, which is the morphological characteristics of BLA principal neurons (McDonald, 1982; Padival et al., 2013). We excluded aspiny neurons showing small somata with few dendrites or large somata with bipolar primary dendrites. A recent study states that the distance from the soma affects the role of inhibitory shaft and spine synapses, and strengthens the role of axon initial segment (Boivin and Nedivi, 2018). Thus, in our study, dendritic segments with 50–150 μm distance from the soma (Christoffel et al., 2011), and 40–70 μm in length, were randomly chosen from pyramidal neurons in the BLA and were counted starting from the origin of a branch. Second-order apical dendrites were analyzed in our study. In order to meet the requirements of spinal analysis, dendritic segments must have the following qualifications: segments must be fully filled (excluding all endings); segments must have a distance of no less than 50 μm from the soma; segments did not show overlap with other branches, which may blur the visualization of spines (Christoffel et al., 2011). A 3D image was reconstructed with NIH ImageJ software^1^. The number of dendritic protrusions were calculated based on the morphology: thin spines have thin head and long neck; mushroom spines come with large head and short neck; stubby spines also have large head but no apparent neck (Montalbano et al., 2013; Geoffroy et al., 2019). For morphological quantification, one dendrite per neuron and 5–8 neurons per rat were analyzed in five rats in each group. The experimenter was blind to the grouping. All images were captured using Olympus BX53 microscope with a 100× oil-immersion objective. The average number of spines per 10 μm of dendrite was calculated.

### Statistical Analysis

Waveform parameters (frequency, amplitude, rise-time 10–90%, half-width, decay time 90–37% and area) (Hendrich et al., 2012) were measured in the study. The results were showed as mean ± SEM. Normal distribution was validated with Shapiro–Wilks test, and homogeneity of variance was validated with Levene’s test. Unpaired Student’s *t*-test was used for comparisons between two groups. Analysis of variance (ANOVA) was used for data analysis with suitable between- and within-subject factors. When comparing three or more groups, one- or two-way ANOVA was adopted with *post hoc* analysis (one-way, Tukey; two-way, Sidak’s multiple comparisons) for comparison of three of more groups. Cumulative probability was compared using Kolmogorov–Smirnov (KS) statistics (P_*KS*_). Since large samples were analyzed, the significance level was mostly taken at *P*_*KS*_ < 0.001 (Simkus and Stricker, 2002; Miura et al., 2012; Calfa et al., 2015).

## Results

**FIGURE 1:**
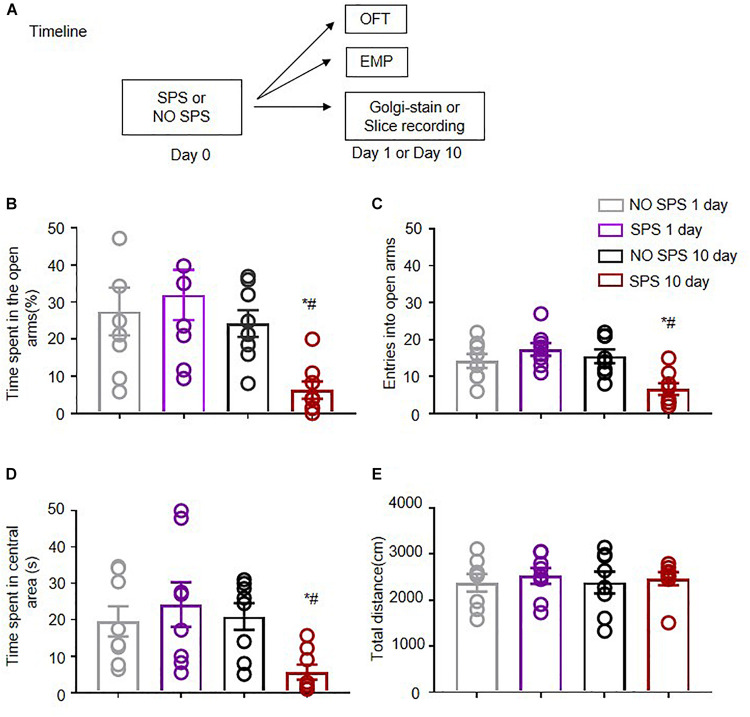
Effect of single prolonged stress (SPS) on anxiety-like behaviors on the first and tenth day after stress. **(A)** Experimental procedures. **(B,C)** Time spent in open arms **(B)** and the entries into open arms **(C)** in different experimental conditions in EPM. **(D,E)** Time spent in the central area **(D)** and total distance (cm) **(E)** in different experimental conditions in OFT. *n* = 8 per experimental condition. ^#^Different from SPS(1d) group, ^∗^Different from NO SPS group at each post-stress day, ^∗^^#^*p* < 0.05, two-way ANOVA. Data are shown as means ± SEM.

Previous investigations have revealed that significant alterations in anxiety-like behaviors occurred only in rats 10 days rather than 1 day after SPS (Fang et al., 2018). Experiment 1 aimed at demonstrating changes in anxiety-like behavior of the rats 1 and 10 days after SPS. Experimental procedure was displayed in Figure 1A. Normal healthy rats were kept separately 4–5 days before the tests to adapt to the feeding environment. Then rats in the experimental group underwent SPS procedure (be restrained for 2 h, forced swimming for 20 min, rest for 15 min and anesthetized with ether until being unconscious), followed by being kept in single cage with undisturbed feeding environment. Anxiety-like behaviors of rats were tested with the EPM and OFT on the first as well as the tenth day after SPS. The experiment mainly consisted of 4 groups: SPS(1d), NO SPS(1d), SPS(10d), NO SPS(10d) (*n* = 8 per group). The results of EPM and OFT were analyzed with two-way ANOVA, and we used SPS (SPS, No SPS) and Post-SPS Day (1 day, 10 day) as the between-subject factors. The analysis of time spent in the open arm in EPM showed significant effects of Post-SPS Day (*F*_1_,_28_ = 7.92, *p* < 0.01) and SPS × Post-SPS Day interaction (*F*_1_,_28_ = 4.76, *p* < 0.05). It was revealed that time spent in the open arm in SPS(10d) group was significantly less compared with NO SPS(10d) group via *post hoc* analysis (*p* < 0.01, Figure 1B). The analysis of entries into the open arms in EPM displayed significant effects of Post-SPS Day (*F*_1_,_28_ = 7.07, *p* < 0.05) and SPS × Post-SPS Day interaction (*F*_1_,_28_ = 11.27, *p* < 0.01). It was revealed that reducing entries were observed in SPS(10d) group in contrast to NO SPS(10d) group via *post hoc* analysis (*p* < 0.01, Figure 1C). The result of time spent in the center area in OFT illustrated significant effects of SPS × Post-SPS Day interaction (*F*_1_,_28_ = 5.45, *p* < 0.05). It was showed that time spent in the central area in SPS(10d) group was obviously less in contrast to the NO SPS(10d) group via *post hoc* analysis (*p* < 0.01, Figure 1D). For total locomotor distance, no obvious difference was shown in experimental conditions (*p* > 0.05, Figure 1E). In summary, these results showed that rats displayed delayed onset of anxiety-like behaviors after SPS, which were in line with previous findings (Knox et al., 2012a; Fang et al., 2018).

**FIGURE 2:**
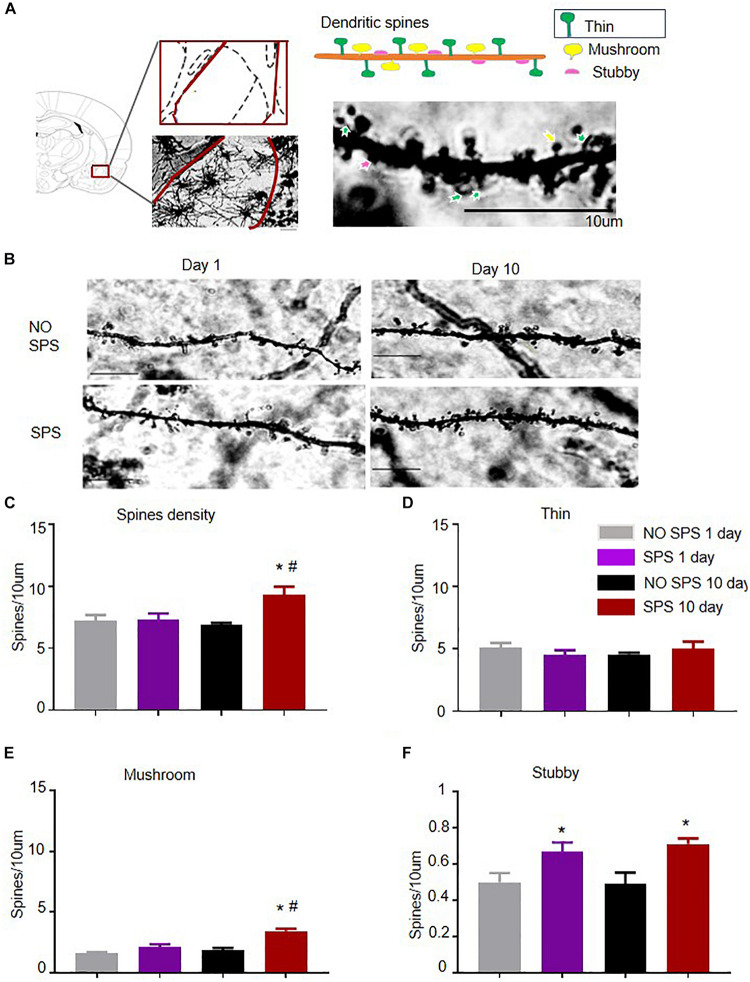
Effect of SPS paradigms on spine density of BLA pyramidal neurons. **(A)** Low-power image of dendritic spines of BLA from SPS-treated rats. Scale bar = 10 μm. Dendritic spines were classified based on morphology: thin dendritic spines have thin head and long neck (indicated by green arrows), mushroom dendritic spines come with large head and short neck (indicated by yellow arrows) and stubby dendritic spines have large head but no apparent neck (indicated by red arrows). Scale bar = 10 μm. **(B)** High-power image of representative dendrite segments (scale bar = 10 μm). **(C)** Spine density in BLA pyramidal dendrite segments in different experimental conditions (animals, rats = 5; segments, *n* = 5–8, total dendritic length = 40–70 μm). **(D–F)** Average density in mushroom **(D)**, thin **(E)**, and stubby **(F)** spines in BLA pyramidal dendrite segments sampled from four groups: NO SPS(1d)/SPS(1d)/NO SPS(10d)/SPS(10d). ^#^Different from SPS(1d) group, ^#*^Different from NO SPS group at each post-SPS day, ^#*^p < 0.05, two-way ANOVA. Data are shown as means ± SEM.

Next, we studied the synaptic mechanisms underlying deferred development of anxiety-like behaviors after SPS. Our previous study showed that activity of BLA glutamatergic neurons and BLA GABAergic neurons was increased day 10 after SPS in contrast to the control group (Fang et al., 2018). BLA is mainly composed of glutamatergic pyramidal neurons (∼85%) (Duvarci and Pare, 2014). Dendrites in the amygdala are especially sensitive to stress exposure (Chattarji et al., 2015). Thus, we analyzed the time-dependent changes in density of dendritic spines in BLA in the SPS model (Figures 2A,B). The experiment consisted of four groups (*n* = 5 per group). Results of the spine density were analyzed with two-way ANOVA, and we used SPS (SPS, No SPS) and Post-SPS Day (1 day, 10 days) as the between-subject factors. The analysis showed noticeable effects of SPS (*F*_1_,_16_ = 6.74, *p* < 0.05) and SPS × Post-SPS Day interaction (*F*_1_,_16_ = 5.73, *p* < 0.05) on the total spine density. The results showed that the density of spines increased obviously in SPS(10d) group in contrast to NO SPS(10d) via *post hoc* analysis (*p* < 0.01, Figure 2C). Dendritic spines are often categorized by morphology and the shape of these spines have correlation with their functions (Moench and Wellman, 2015). Thus, we analyzed the spine densities of different subtypes (Wang et al., 2017) in BLA after SPS. Analysis with two-way ANOVA revealed noticeable effects of SPS (*F*_1_,_16_ = 29.60, *p* < 0.01), Post-SPS Day (*F*_1_,_16_ = 17.22, *p* < 0.01) and SPS × Post-SPS Day interaction (*F*_1_,_16_ = 7.76, *p* < 0.05) on the density of mushroom spines, but the analysis on the density of thin spines revealed no significant effects (*p* > 0.05). *Post hoc* analysis showed that the density of mushroom spines increased remarkably in the SPS(10d) group (*p* < 0.05, Figure 2E), while no significant differences were found in the SPS(1d) group. The density of thin spines showed no significant difference in the SPS(1d) group or SPS(10d) group (*p* > 0.05, Figure 2D). Density of stubby spines, which were reckoned to be immature structures and had a certain relationship with the stress-induced increase of glutamatergic synapses, was increased both of day 1 and day 10 after SPS in contrast to corresponding control groups (both *p* < 0.05, Figure 2F) (Christoffel et al., 2011). To sum up, total and mushroom spine density were markedly increased in SPS(10d) group in our research, which accompanied an increase in anxiety-like behavior in SPS(10d) group.

**FIGURE 3:**
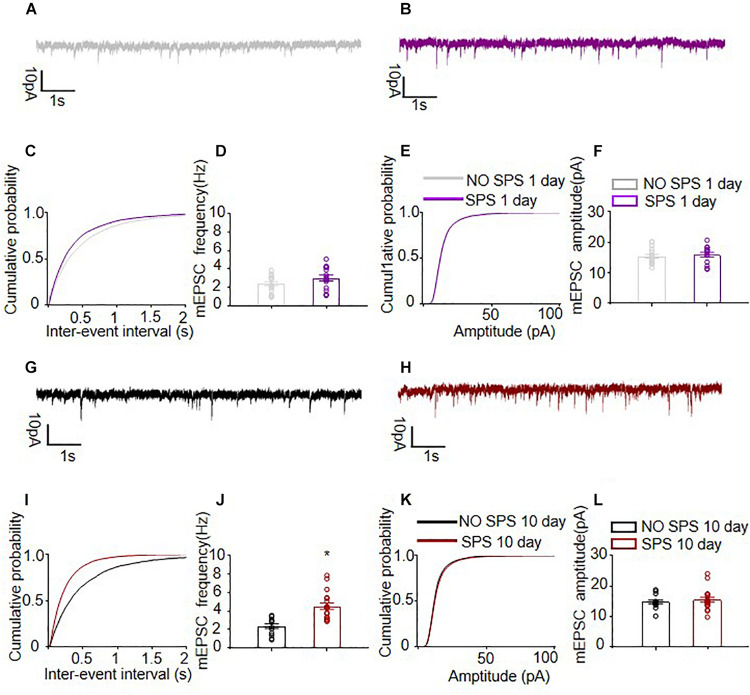
Spontaneous excitatory quantal synaptic transmission onto BLA pyramidal neurons. **(A,B,G,H)** Representative examples of mEPSCs of different experimental conditions. **(C,I)** The cumulative probability distribution of mEPSCs inter-event intervals of BLA pyramidal neurons from SPS group and NO SPS group on the first and tenth day after SPS respectively. **(D,J)** The mEPSCs frequency of BLA pyramidal neurons from SPS group and NO SPS group on the first and tenth day after SPS respectively. **(E,K)** The cumulative probability distribution of mEPSCs amplitudes of BLA pyramidal neurons from SPS group and NO SPS group on the first and tenth day after SPS respectively. **(F,L)** The mEPSCs amplitudes of BLA pyramidal neurons from SPS group and NO SPS group on the first and tenth day after SPS respectively. ^∗^Different from NO SPS group at each post-SPS day, ^∗^*p* < 0.05, one-way ANOVA. Data are shown as means ± SEM.

**FIGURE 4:**
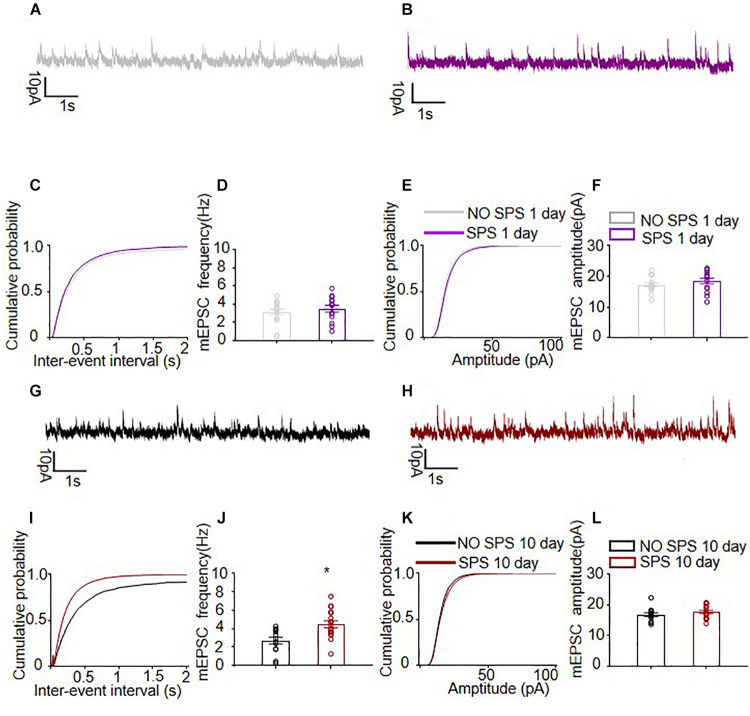
Spontaneous inhibitory quantal synaptic transmission onto BLA pyramidal neurons. **(A,B,G,H)** Representative examples of mIPSCs of different experimental conditions. **(C,I)** The cumulative probability distribution of mIPSCs inter-event intervals of BLA pyramidal neurons from SPS group and NO SPS group on the first and tenth day after SPS respectively. **(D,J)** The mIPSCs frequency of BLA pyramidal neurons from rats on SPS group and NO SPS group. **(E,K)** The cumulative probability distribution of mIPSCs amplitudes of BLA pyramidal neurons from SPS group and NO SPS group on the first and tenth day after SPS respectively. **(F,L)** The mIPSCs amplitudes of BLA pyramidal neurons from rats on SPS group and NO SPS group on the first and tenth day after SPS respectively. ^∗^Different from NO SPS group at each post-SPS day, ^∗^*p* < 0.05, one-way ANOVA. Data are shown as means ± SEM.

Formation and elimination of dendritic spines may contribute to synaptic connectivity and function, especially mushroom spines positively correlating with synapse strength and age (Holtmaat and Svoboda, 2009; Moench and Wellman, 2015). Therefore, following the same SPS procedure, we recorded mIPSCs and mEPSCs of the same cell at different voltages in the SPS(1d) and SPS(10d) group, respectively to determine spontaneous quantal synaptic input onto BLA pyramidal neurons (Figures 3, 4). For the input resistance, there was no significant difference among experimental conditions (*p* > 0.05, Supplementary Figure S2A). Data of the frequency and amplitude of mEPSCs and mIPSCs were analyzed with one-way ANOVA, and we used SPS as the between-subjects factor (*n* = 13–18 cells per group). The mEPSCs frequency recorded in BLA pyramidal neurons of the SPS(10d) group was remarkably increased compared with the NO SPS group (*F*_1_,_2__9_ = 20.93, *p* < 0.01, Figure 3J), while no obvious difference was found in the day 1 group after SPS (*p* > 0.05, Figure 3D). For mEPSCs inter-event intervals of BLA pyramidal neurons, the cumulative probability distribution of SPS(10d) group was shifted left compared with NO SPS group (*P*_*KS*_ < 0.001, Figure 3I), which indicated that mEPSCs frequency in SPS(10d) group was increased. The cumulative probability distribution was mildly shifted toward the left (*P*_*KS*_ = 0.0015, Figure 3C), which may be due to a slight increase in mEPSCs frequency in SPS(1d) group. The mEPSCs amplitudes recorded in BLA pyramidal neurons of the SPS(1d) group as well as SPS(10d) group showed no significant difference with NO SPS groups (both *p* > 0.05, Figures 3F,L). The cumulative probability distribution of amplitudes of mEPSCs in SPS(1d) and SPS(10d) group BLA pyramidal neurons were not shifted compared to NO SPS pyramidal neurons (both *p* > 0.05, Figures 3E,K). Finally, we compared the amplitude and frequency of mEPSCs in BLA pyramidal neurons of the SPS(1d) group and SPS(10d) group. The excitatory synaptic frequency of pyramidal neurons of the SPS(10d) group was increased compared with that of the SPS(1d) group (*F*_1_,_3__0_ = 9.18, *p* < 0.01, Table 1), and no difference was observed for the amplitude (*p* > 0.05, Table 1). The results showed that the amplitude of mEPSCs of BLA pyramidal neurons after SPS was not obviously affected, but frequency of mEPSCs in BLA pyramidal neurons increased significantly day 10 after SPS. Finally, as larger spines often predict larger mEPSC amplitude (Segal, 2010; Ueno et al., 2014), we analyzed the mEPSCs after classifying spikes into different subgroups by different amplitude values (Lu et al., 2007; Biggs et al., 2010). One-way ANOVA analyzed the large amplitude events (>30 pA) of mEPSCs in BLA pyramidal neurons from four groups, the results showed that the large amplitude events (>30 pA) of mEPSCs in BLA pyramidal neurons of the SPS(10d) group was increased compared with control groups (*F*_3_,_45_ = 4.16, *p* < 0.05, Supplementary Figure S2B). In summary, the results showed that the excitatory synaptic transmission of BLA pyramidal neurons increased day 10 after SPS.

**TABLE 1:** Summary of electrophysiological data.

**Group**		**Frequency (Hz)**	**Amplitude (pA)**	**Risetime 10–90% (ms)**	**Half-width (ms)**	**Decay 90–37%(ms)**
		**Mean ± sem**	**Mean ± sem**	**Mean ± sem**	**Mean ± sem**	**Mean ± sem**
NO SPS (1d)	mEPSCs	2.41 ± 0.22, *n* = 18	15.41 ± 0.59, *n* = 18	1.09 ± 0.05, *n* = 18	6.48 ± 0.15, *n* = 18	5.79 ± 0.26, *n* = 18
	mIPSCs	3.17 ± 0.28, *n* = 18	17.29 ± 0.57, *n* = 18	1.42 ± 0.19, *n* = 18	14.32 ± 0.43, *n* = 18	13.83 ± 0.52, *n* = 18
SPS (1d)	mEPSCs	3.03 ± 0.36, *n* = 14	15.87 ± 0.78, *n* = 14	1.00 ± 0.05, *n* = 14	5.37 ± 0.37, *n* = 14	4.83 ± 0.24, *n* = 14
	mIPSCs	3.52 ± 0.37, *n* = 14	18.53 ± 0.93, *n* = 14	1.34 ± 0.21, *n* = 14	13 ± 0.59, *n* = 14	12.8 ± 0.65, *n* = 14
NO SPS (10d)	mEPSCs	2.38 ± 0.25, *n* = 13	14.88 ± 0.64, *n* = 13	1.08 ± 0.06, *n* = 13	5.85 ± 0.38, *n* = 13	5.18 ± 0.33, *n* = 13
	mIPSCs	2.71 ± 0.37, *n* = 13	16.84 ± 0.62, *n* = 13	1.13 ± 0.21, *n* = 13	12.8 ± 0.51, *n* = 13	14.08 ± 0.59, *n* = 13
SPS (10d)	mEPSCs	4.53 ± 0.35^∗^#, *n* = 18	15.56 ± 0.82, *n* = 18	1.10 ± 0.07, *n* = 18	5.91 ± 0.33, *n* = 18	5.07 ± 0.27, *n* = 18
	mIPSCs	4.48 ± 0.35^∗^, *n* = 18	17.68 ± 0.51, *n* = 18	1.26 ± 0.15, *n* = 18	12.7 ± 0.54, *n* = 18	12.31 ± 0.47, *n* = 18

*^∗^Different from NO SPS group at each post-SPS day, ^#^Different from SPS(1d) group, *p* < 0.05, one-way ANOVA. Data are shown as means ± SEM.*

We further recorded the mIPSCs of the same pyramidal neurons paired with mEPSCs after the SPS procedure (Calfa et al., 2015) with 1 μM TTX (Figure 4) at 0 mV (*n* = 13–18 cells per group). The mIPSCs frequency recorded in BLA pyramidal neurons 1 day after SPS exhibited no changes compared with controls (*p* > 0.05, Figure 4D), while the SPS(10d) group showed significantly higher mIPSCs frequency than control groups (*F*_1_,_29_ = 11.60, *p* < 0.01, Figure 3J). For mIPSCs inter-event intervals of BLA pyramidal neurons, the cumulative probability distribution of the SPS(10d) group was shifted left compared with that of NO SPS pyramidal neurons (*P*_*KS*_ < 0.001, Figure 4I), while no difference was found between the SPS(1d) group and NO SPS group. On the other hand, the mIPSCs amplitude recorded in BLA pyramidal neurons of either SPS(1d) or SPS(10d) group showed no difference compared with NO SPS groups (both *p* > 0.05, Figures 4F,L). In BLA pyramidal neurons, the curves of the cumulative probability distribution of mIPSCs amplitude from SPS(1d) group and SPS(10d) group were not shifted compared with control pyramidal neurons and they almost coincided (both *p* > 0.05, Figures 4E,K). Then, we compared the amplitude and frequency of mIPSCs in BLA pyramidal neurons of SPS(1d) group and SPS(10d) group, and the analysis showed that the inhibitory synaptic frequency of BLA pyramidal neurons of the SPS(10d) group was obviously higher compared to that of the SPS(1d) group (*F*_1_,_30_ = 3.51, *p* > 0.05, Table 1), and no changes were found in amplitude (*p* > 0.05, Table 1). In summary, the results showed that the inhibitory synaptic transmission of BLA pyramidal neurons increased day 10 after SPS. The results showed that BLA pyramidal neurons received enhanced inhibitory neuronal projections day 10 after SPS, and the frequency of mEPSCs and mIPSCs were increased.

## Discussion

Patients with PTSD typically have symptoms such as avoidance, interference and awakening, emotional and cognitive changes (Pitman et al., 2012). Extensive reports have used SPS procedure to study the animal PTSD (Iwamoto et al., 2007; Wen et al., 2016; Fang et al., 2018). Research has revealed that SPS leads to diminished fear extinction (Knox et al., 2012a; Fang et al., 2018), enhanced stress-induced nociceptive sensitivity and increased anxiety-like behavior (Zhang et al., 2012), and SSRI may reverse the symptoms (Takahashi et al., 2006; Lin et al., 2016). We assessed PTSD-induced anxiety-like behavior through OFT and EPM. Our behavioral experiments revealed no notable alterations in the anxiety-like behavior of rats on the first day after SPS, but a significant increase consistent with previous findings on the 10th day after SPS (Fang et al., 2018). Furthermore, we found delayed changes in synaptic plasticity in BLA pyramidal neurons after SPS. Specifically, on day 10 after exposure to SPS, result indicated an increase in density of dendritic spine, and enhancement both in glutamatergic and GABAergic synaptic transmissions. In conclusion, SPS produced delayed increase in spinogenesis and synaptic transmission in BLA which is accompanied with enhanced anxiety-like behaviors.

The structural plasticity of dendritic spines is critical for diverse types of synaptic plasticity (Yang et al., 2009; Yin et al., 2009; Oe et al., 2013), including structural remodeling in response to stress (Chattarji et al., 2015; Duman and Duman, 2015; Qiao et al., 2016). The structural basis of synaptic connectivity in BLA is differentially modified by various forms of stress (Chattarji et al., 2015). Acute restraint stress induces an enhancement in dendritic spine density in the BLA pyramidal neurons several days after stress (Mitra et al., 2005; Maroun et al., 2013; Suvrathan et al., 2014; Yasmin et al., 2016). Chronic restraint stress induces dendritic hypertrophy in BLA pyramidal neurons, increased size of dendritic spine heads (Mitra et al., 2005; Vyas et al., 2006; Maroun et al., 2013; Zhang et al., 2019) and enhanced neuronal excitability (Rosenkranz et al., 2010). Consistently, our results showed that dendritic spine density in BLA pyramidal neurons of SPS(10d) group was increased. However, a recent study showed that acute elevated platform stress increased mushroom spine density and produced dendritic retraction in BLA pyramidal neurons 2 days later (Maroun et al., 2013). We presumed that the discrepancies between the findings on effects of stress on dendritic morphology of amygdala may be due to different types and procedures of stress. The present results showed that mushroom spines but not thin spines displayed delayed increase after SPS. Generally, thin spines have higher plasticity and lability compared with mushroom spines (Moench and Wellman, 2015). Thus, our results suggest that the mature and stable type of spines are gradually increased after traumatic stress which may be the structural substrates of delayed onset of anxiety-like behaviors. While the mechanisms underlying the delayed alteration of dendritic spines remain unclear, it is worthy to note the implications of NMDA and AMPA receptors in regulating structural plasticity (Krugers et al., 2010; Duman, 2014; Yasmin et al., 2016). NMDA receptors are considered to be implicated in the initial formation of spines by calcium influx and continuous downstream effects (Maletic-Savatic et al., 1999), and AMPA receptors are implicated in the strengthening of existing spines (Maletic-Savatic et al., 1999). In the amygdala, 10 days of chronic immobilization stress could enhance NMDAR-mediated synaptic responses (Suvrathan et al., 2014), and the NMDAR antagonist infused into the BLA during the acute stress prevented the enhanced effects on mEPSCs frequency and spine density 10 days later (Yasmin et al., 2016). It has been demonstrated that the ratio of GluA1-AMPAR-labeled spines to labeled dendritic shafts in the BLA was found to increase 6 and 14 days but not 1 day after stress, which accompanies enhanced frequency of mEPSCs in stressed animals without changes in mEPSCs amplitude (Hubert et al., 2014). Thus, we speculated that AMPA receptors are associated with the expression and maintenance of stress-induced structural plasticity, while NMDA receptors are important for the initiation of stress-induced structural plasticity. Interestingly, we found the stubby spines were increased on both day 1 and 10 after traumatic stress. A stubby spine with a large head and no neck is considered as a type of immature spines (Ebrahimi and Okabe, 2014; Berry and Nedivi, 2017). Although stubby structures are rarely studied and understood, it is reported that they predominate early in postnatal development (Boyer et al., 1998) and to proliferate in nucleus accumbens after social stress (Christoffel et al., 2011). Considering the roles of the geometry of the spine neck in synaptic plasticity, stubby spines may elicit strong signal diffusing through the surrounding dendrite (Hayashi and Majewska, 2005; Ebrahimi and Okabe, 2014), which may be involved in anxiety-like behaviors. The precise roles of stubby spines in the amygdala structural plasticity and maladaptive response to stress need to be further investigated.

Spines are important targets for excitatory synaptic transmission (Harris and Kater, 1994; Qiao et al., 2016) and are positively associated with synaptic transmission (Hayashi and Majewska, 2005; Alvarez and Sabatini, 2007; Ebrahimi and Okabe, 2014). In our current study, the analysis of the mEPSCs frequency showed a delayed enhancement in BLA after SPS, which is consistent with the findings that an increased number of excitatory pyramidal neurons were activated on the 10th day after SPS (Fang et al., 2018). Consistently, Yasmin and colleagues found that increase in mEPSCs frequency induced by stress is associated with an enhancement of the number of dendritic spines (Yasmin et al., 2016). Escalation in the frequency of mEPSCs is considered to be due to an increase in the number of glutamatergic synapses and a presynaptic suppression of glutamate release probability (Malgaroli and Tsien, 1992; Sastry and Bhagavatula, 1996). Considering the significant increase in the number of dendritic spines in BLA in SPS(10d) group, the observed enhancement of mEPSCs frequency in our study may be induced, at least in part, by the increase in the number of functional excitatory synapses. Under some circumstances, some other studies have reported that an increase in the number of dendrites spines accompanies an enhancement of frequency of mEPSCs (Wissman et al., 2011; Montalbano et al., 2013; Bochner et al., 2014; Yasmin et al., 2016; Schilling et al., 2017; Sun et al., 2018). However, the increased number of spines, especially large spines, would be predictive of an increase in the expression of postsynaptic excitatory receptors and subsequently larger mEPSCs amplitude (Lee et al., 2015; Udagawa et al., 2015; Awad et al., 2016; Deng et al., 2019). Consistently, we showed that the amplitude (>30 pA) of mEPSCs in BLA pyramidal neurons of SPS(10d) group was increased compared with No SPS groups, which fits with the observed increasing density of mushroom spines on the 10th day after SPS.

Interestingly, we found an enhancement in the frequency of inhibitory synaptic transmission 10 days after stress. Combined with our previous findings that more inhibitory neurons are activated on the 10th day after SPS (Fang et al., 2018), we considered that also gradually activated inhibitory neurons which would be due to either an increase in the number of GABAergic synapses or an increase in the release probability. More data, such as spontaneous IPSC are required to confirm these explanations in the future. It is essential to explore the effects of inhibitory transmission on stress-induced BLA dysfunction and delayed appearance of PTSD-like behaviors. It has been shown that function of adult BLA is regulated by a reciprocal interaction between GABAergic interneurons and pyramidal neurons (Ehrlich et al., 2009, 2012; Ryan et al., 2012), so the delayed increase in inhibitory transmission may be attributed to a homeostatic mechanism which avoids excessive activation of the pyramidal neurons in BLA. The current finding was in line with previous results that chronic activity blockade leads to homeostatic plasticity that both mEPSCs and mIPSCs frequency were elevated (Echegoyen et al., 2007). We found that the frequency of IPSCs and EPSCs increase by similar amounts after stress, and the balance between inhibition and excitation seems to be unaltered. We presumed that other cellular and synaptic mechanisms may also contribute to the PTSD-like behaviors in rats, such as the alterations in a specific type of GABAergic neurons in BLA after traumatic stress or time-dependent distributions of inhibitory synapse on pyramidal neurons after stress. Furthermore, it is unclear if the excitability of pyramidal cells or activity-dependent network plasticity would be significantly altered. Further experiments investigating the effects of stress upon intrinsic excitability, spontaneous EPSCs/IPSCs and evoked EPSCs/IPSCs would be informative in this regard. Lastly, it should be noted, with various corticolimbic targets, that BLA pyramidal neurons are functionally heterogeneous and thus stress may differentially impact specific output circuits. Indeed, dendrites were hypertrophied caused by chronic restraint stress in BLA pyramidal neurons, and the size of dendritic spine heads was increased only in BLA pyramidal neurons targeting the nucleus accumbens (NAc) or the ventral hippocampus (vHPC) (Zhang et al., 2019). In addition, the excitatory glutamatergic transmission targeting the vHPC or the NAc in BLA PNs was selectively increased (Zhang et al., 2019). Therefore, which BLA projects exhibit changes of excitation-inhibition balance after SPS needs to be further investigated.

The underlying molecular mechanism of delayed increase in spine density and neural transmissions is still unknown, and previous evidence suggests that it may be related to dysregulation of the HPA axis, with significant lower concentrations of plasma and urinary cortisol (Yehuda et al., 1993). Previous studies speculated that hypercortisol and glucocorticoid negative feedback is specifically increased by PTSD (Zoladz and Diamond, 2013). Consistently, it has been shown that the delayed spinogenesis in the BLA can be impeded by prior exposure to glucocorticoids after acute stress, which could be blocked by bilateral adrenalectomy (Rao et al., 2012). Furthermore, some studies have revealed that SPS increases the expression level of glucocorticoid receptors (Ganon-Elazar and Akirav, 2013), and NMDA receptor subunit mRNAs (Yamamoto et al., 2008). However, another study showed that the expression level of CaMKII and MR/GR in BLA had not been obviously affected by SPS, and the improvement of NPY functions could regulate the alterations in the morphology of the BLA pyramidal neurons induced by SPS (Cui et al., 2008). Thus, more research is required to discover the molecular mechanisms of the increase in spinogenesis and synaptic transmission after SPS.

The results of present study revealed that rats showed increase in both spinogenesis and synaptic transmission in the BLA only on day 10 rather than day 1 after SPS, which means after traumatic stress, BLA displayed delayed changes in neuronal plasticity. The present findings revealed that BLA may be associated with the pathogenesis of PTSD, which is of great importance for future clinical research and targeted treatment.

## Data Availability Statement

All datasets generated for this study are included in the article/Supplementary Material.

## Ethics Statement

All experiments were performed in accordance with the National Institutes of Health Guide for the Care and Use of Laboratory Animals and Biomedical Ethics Committee of Peking University for animal use and protection. The protocol was approved by the Biomedical Ethics Committee of Peking University for animal use and protection.

## Author Contributions

H-HZ, S-QM, J-LY, and Y-XX designed the experiments. H-HZ, S-QM, X-YG, and Y-YC performed the experiments. H-HZ and Y-XX analyzed and interpreted the data. J-LZ, WZ, S-QM, and J-LY commented on the manuscript. H-HZ, J-LZ, Y-XX, and LL wrote the manuscript.

## Conflict of Interest

The authors declare that the research was conducted in the absence of any commercial or financial relationships that could be construed as a potential conflict of interest.

## Footnotes

1
http://rsbweb.nih.gov/ij/

## Supplementary Material

FIGURE S1 **(A)** Experimental timeline. **(B)** In defect of antagonists of glutamatergic or GABAergic receptors, mEPSCs and mIPSCs were recorded at clamped voltages of −70 and 0 mV. After adding 20 μM CNQX and 50 μM AP5, the postsynaptic currents recorded at −70 mV were blocked, while those recorded at 0 mV were suppressed by 50 μM picrotoxin.

FIGURE S2 **(A)** The input resistance of BLA pyramidal neurons among the different experimental conditions. **(B)** Large amplitude events (>30 pA) of mEPSCs in BLA pyramidal neurons from four groups: NO SPS(1d)/SPS(1d)/NO SPS(10d)/SPS(10d). ^∗^Different from NO SPS groups. One-way ANOVA, ^∗^*p* < 0.05.

## References

[B1] Abuhasan Q. Siddiqui W. (2019). *Neuroanatomy, Amygdala.* Treasure Island, FL: StatPearls Publishing. 30725787

[B2] Alvarez V. A. Sabatini B. L. (2007). Anatomical and physiological plasticity of dendritic spines. *Annu. Rev. Neurosci.* 30 79–97. 10.1146/annurev.neuro.30.051606.094222 17280523

[B3] Awad P. N. Sanon N. T. Chattopadhyaya B. Carrico J. N. Ouardouz M. Gagne J. (2016). Reducing premature KCC2 expression rescues seizure susceptibility and spine morphology in atypical febrile seizures. *Neurobiol. Dis.* 91 10–20. 10.1016/j.nbd.2016.02.014 26875662

[B4] Bailey C. R. Cordell E. Sobin S. M. Neumeister A. (2013). Recent progress in understanding the pathophysiology of post-traumatic stress disorder: implications for targeted pharmacological treatment. *CNS Drugs* 27 221–232. 10.1007/s40263-013-0051-4 23483368 PMC3629370

[B5] Berdel B. Morys J. Maciejewska B. (1997). Neuronal changes in the basolateral complex during development of the amygdala of the rat. *Int. J. Dev. Neurosci.* 15 755–765. 10.1016/S0736-5748(97)00022-1 9402226

[B6] Berry K. P. Nedivi E. (2017). Spine dynamics: are they all the same? *Neuron* 96 43–55. 10.1016/j.neuron.2017.08.008 28957675 PMC5661952

[B7] Biggs J. E. Lu V. B. Stebbing M. J. Balasubramanyan S. Smith P. A. (2010). Is BDNF sufficient for information transfer between microglia and dorsal horn neurons during the onset of central sensitization? *Mol Pain* 6:44. 10.1186/1744-8069-6-44 20653959 PMC2918544

[B8] Bochner D. N. Sapp R. W. Adelson J. D. Zhang S. Lee H. Djurisic M. (2014). Blocking PirB up-regulates spines and functional synapses to unlock visual cortical plasticity and facilitate recovery from amblyopia. *Sci. Transl. Med.* 6:258ra140. 10.1126/scitranslmed.3010157 25320232 PMC4476552

[B9] Boivin J. R. Nedivi E. (2018). Functional implications of inhibitory synapse placement on signal processing in pyramidal neuron dendrites. *Curr. Opin. Neurobiol.* 51 16–22. 10.1016/j.conb.2018.01.013 29454834 PMC6066407

[B10] Bourne J. Harris K. M. (2007). Do thin spines learn to be mushroom spines that remember? *Curr. Opin. Neurobiol.* 17 381–386. 10.1016/j.conb.2007.04.009 17498943

[B11] Boyer C. Schikorski T. Stevens C. F. (1998). Comparison of hippocampal dendritic spines in culture and in brain. *J. Neurosci.* 18 5294–5300. 10.1523/JNEUROSCI.18-14-05294.1998 9651212 PMC6793498

[B12] Bradley R. Greene J. Russ E. Dutra L. Westen D. (2005). A multidimensional meta-analysis of psychotherapy for PTSD. *Am. J. Psychiatry* 162 214–227. 10.1176/appi.ajp.162.2.214 15677582

[B13] Bryant R. A. Felmingham K. Kemp A. Das P. Hughes G. Peduto A. (2008). Amygdala and ventral anterior cingulate activation predicts treatment response to cognitive behaviour therapy for post-traumatic stress disorder. *Psychol. Med.* 38 555–561. 10.1017/S0033291707002231 18005496

[B14] Calfa G. Li W. Rutherford J. M. Pozzo-Miller L. (2015). Excitation/inhibition imbalance and impaired synaptic inhibition in hippocampal area CA3 of Mecp2 knockout mice. *Hippocampus* 25 159–168. 10.1002/hipo.22360 25209930 PMC4300269

[B15] Chattarji S. Tomar A. Suvrathan A. Ghosh S. Rahman M. M. (2015). Neighborhood matters: divergent patterns of stress-induced plasticity across the brain. *Nat. Neurosci.* 18 1364–1375. 10.1038/nn.4115 26404711

[B16] Christoffel D. J. Golden S. A. Dumitriu D. Robison A. J. Janssen W. G. Ahn H. F. (2011). IkappaB kinase regulates social defeat stress-induced synaptic and behavioral plasticity. *J. Neurosci.* 31 314–321. 10.1523/JNEUROSCI.4763-10.2011 21209217 PMC3219041

[B17] Cohen B. E. Gima K. Bertenthal D. Kim S. Marmar C. R. Seal K. H. (2010). Mental health diagnoses and utilization of VA non-mental health medical services among returning Iraq and Afghanistan veterans. *J. Gen. Intern. Med.* 25 18–24. 10.1007/s11606-009-1117-3 19787409 PMC2811589

[B18] Cohen H. Kozlovsky N. Matar M. A. Zohar J. Kaplan Z. (2014). Distinctive hippocampal and amygdalar cytoarchitectural changes underlie specific patterns of behavioral disruption following stress exposure in an animal model of PTSD. *Eur. Neuropsychopharmacol.* 24 1925–1944. 10.1016/j.euroneuro.2014.09.009 25451698

[B19] Cohen S. Kozlovsky N. Matar M. A. Kaplan Z. Zohar J. Cohen H. (2012). Post-exposure sleep deprivation facilitates correctly timed interactions between glucocorticoid and adrenergic systems, which attenuate traumatic stress responses. *Neuropsychopharmacology* 37 2388–2404. 10.1038/npp.2012.94 22713910 PMC3442354

[B20] Cui H. Sakamoto H. Higashi S. Kawata M. (2008). Effects of single-prolonged stress on neurons and their afferent inputs in the amygdala. *Neuroscience* 152 703–712. 10.1016/j.neuroscience.2007.12.028 18308474

[B21] Deng Z. F. Zheng H. L. Chen J. G. Luo Y. Xu J. F. Zhao G. (2019). miR-214-3p targets beta-catenin to regulate depressive-like behaviors induced by chronic social defeat stress in mice. *Cereb. Cortex* 29 1509–1519. 10.1093/cercor/bhy047 29522177

[B22] Dias B. G. Goodman J. V. Ahluwalia R. Easton A. E. Andero R. Ressler K. J. (2014). Amygdala-dependent fear memory consolidation via miR-34a and Notch signaling. *Neuron* 83 906–918. 10.1016/j.neuron.2014.07.019 25123309 PMC4172484

[B23] Ding J. Han F. Shi Y. (2010). Single-prolonged stress induces apoptosis in the amygdala in a rat model of post-traumatic stress disorder. *J. Psychiatr. Res.* 44 48–55. 10.1016/j.jpsychires.2009.06.001 19586638

[B24] Ding J. Han F. Wen L. Xiao B. Shi Y. (2017). The role of beta-arrestin-2 on Fear/anxious-related memory in a rat model of Post-traumatic stress disorder. *J. Affect. Disord.* 213 1–8. 10.1016/j.jad.2016.12.043 28167453

[B25] Duman C. H. Duman R. S. (2015). Spine synapse remodeling in the pathophysiology and treatment of depression. *Neurosci. Lett.* 601 20–29. 10.1016/j.neulet.2015.01.022 25582786 PMC4497940

[B26] Duman R. S. (2014). Neurobiology of stress, depression, and rapid acting antidepressants: remodeling synaptic connections. *Depress. Anxiety* 31 291–296. 10.1002/da.22227 24616149 PMC4432471

[B27] Duvarci S. Pare D. (2014). Amygdala microcircuits controlling learned fear. *Neuron* 82 966–980. 10.1016/j.neuron.2014.04.042 24908482 PMC4103014

[B28] Ebrahimi S. Okabe S. (2014). Structural dynamics of dendritic spines: molecular composition, geometry and functional regulation. *Biochim. Biophys. Acta* 1838 2391–2398. 10.1016/j.bbamem.2014.06.002 24915021

[B29] Echegoyen J. Neu A. Graber K. D. Soltesz I. (2007). Homeostatic plasticity studied using in vivo hippocampal activity-blockade: synaptic scaling, intrinsic plasticity and age-dependence. *PLoS One* 2:e700. 10.1371/journal.pone.0000700 17684547 PMC1933594

[B30] Ehrlich D. E. Ryan S. J. Rainnie D. G. (2012). Postnatal development of electrophysiological properties of principal neurons in the rat basolateral amygdala. *J. Physiol.* 590 4819–4838. 10.1113/jphysiol.2012.237453 22848043 PMC3487039

[B31] Ehrlich I. Humeau Y. Grenier F. Ciocchi S. Herry C. Luthi A. (2009). Amygdala inhibitory circuits and the control of fear memory. *Neuron* 62 757–771. 10.1016/j.neuron.2009.05.026 19555645

[B32] Fang Q. Li Z. Huang G. D. Zhang H. H. Chen Y. Y. Zhang L. B. (2018). Traumatic stress produces distinct activations of GABAergic and glutamatergic neurons in Amygdala. *Front. Neurosci.* 12:387. 10.3389/fnins.2018.00387 30186100 PMC6110940

[B33] Galea S. Brewin C. R. Gruber M. Jones R. T. King D. W. King L. A. (2007). Exposure to hurricane-related stressors and mental illness after Hurricane Katrina. *Arch. Gen. Psychiatry* 64 1427–1434. 10.1001/archpsyc.64.12.1427 18056551 PMC2174368

[B34] Ganon-Elazar E. Akirav I. (2013). Cannabinoids and traumatic stress modulation of contextual fear extinction and GR expression in the amygdala-hippocampal-prefrontal circuit. *Psychoneuroendocrinology* 38 1675–1687. 10.1016/j.psyneuen.2013.01.014 23433741

[B35] Geoffroy H. Canestrelli C. Marie N. Noble F. (2019). Morphine-Induced dendritic Spine remodeling in rat nucleus accumbens is corticosterone dependent. *Int. J. Neuropsychopharmacol.* 22 394–401. 10.1093/ijnp/pyz014 30915438 PMC6545536

[B36] Gipson C. D. Olive M. F. (2017). Structural and functional plasticity of dendritic spines - root or result of behavior? *Genes Brain Behav.* 16 101–117. 10.1111/gbb.12324 27561549 PMC5243184

[B37] Han F. Ding J. Shi Y. (2014). Expression of amygdala mineralocorticoid receptor and glucocorticoid receptor in the single-prolonged stress rats. *BMC Neurosci.* 15:77. 10.1186/1471-2202-15-77 24947040 PMC4074391

[B38] Han K. S. Woo J. Park H. Yoon B. J. Choi S. Lee C. J. (2013). Channel-mediated astrocytic glutamate release via Bestrophin-1 targets synaptic NMDARs. *Mol. Brain* 6:4. 10.1186/1756-6606-6-4 23324492 PMC3577500

[B39] Han Y. Luo Y. Sun J. Ding Z. Liu J. Yan W. (2016). AMPK signaling in the dorsal hippocampus negatively regulates contextual fear memory formation. *Neuropsychopharmacology* 41 1849–1864. 10.1038/npp.2015.355 26647974 PMC4869054

[B40] Harris K. M. Kater S. B. (1994). Dendritic spines: cellular specializations imparting both stability and flexibility to synaptic function. *Annu. Rev. Neurosci.* 17 341–371. 10.1146/annurev.ne.17.030194.002013 8210179

[B41] Hayashi Y. Majewska A. K. (2005). Dendritic spine geometry: functional implication and regulation. *Neuron* 46 529–532. 10.1016/j.neuron.2005.05.006 15944122

[B42] Hendrich J. Bauer C. S. Dolphin A. C. (2012). Chronic pregabalin inhibits synaptic transmission between rat dorsal root ganglion and dorsal horn neurons in culture. *Channels* 6 124–132. 10.4161/chan.19805 22627148 PMC3396689

[B43] Holtmaat A. Svoboda K. (2009). Experience-dependent structural synaptic plasticity in the mammalian brain. *Nat. Rev. Neurosci.* 10 647–658. 10.1038/nrn2699 19693029

[B44] Hubert G. W. Li C. Rainnie D. G. Muly E. C. (2014). Effects of stress on AMPA receptor distribution and function in the basolateral amygdala. *Brain Struct. Funct.* 219 1169–1179. 10.1007/s00429-013-0557-z 23644586 PMC3884034

[B45] Hughes K. C. Shin L. M. (2011). Functional neuroimaging studies of post-traumatic stress disorder. *Expert Rev. Neurother.* 11 275–285. 10.1586/ern.10.198 21306214 PMC3142267

[B46] Iwamoto Y. Morinobu S. Takahashi T. Yamawaki S. (2007). Single prolonged stress increases contextual freezing and the expression of glycine transporter 1 and vesicle-associated membrane protein 2 mRNA in the hippocampus of rats. *Prog. Neuropsychopharmacol. Biol. Psychiatry* 31 642–651. 10.1016/j.pnpbp.2006.12.010 17267088

[B47] Keyes K. M. McLaughlin K. A. Demmer R. T. Cerda M. Koenen K. C. Uddin M. (2013). Potentially traumatic events and the risk of six physical health conditions in a population-based sample. *Depress. Anxiety* 30 451–460. 10.1002/da.22090 23495094 PMC4180235

[B48] Knox D. George S. A. Fitzpatrick C. J. Rabinak C. A. Maren S. Liberzon I. (2012a). Single prolonged stress disrupts retention of extinguished fear in rats. *Learn. Mem.* 19 43–49. 10.1101/lm.024356.111 22240323 PMC3262971

[B49] Knox D. Nault T. Henderson C. Liberzon I. (2012b). Glucocorticoid receptors and extinction retention deficits in the single prolonged stress model. *Neuroscience* 223 163–173. 10.1016/j.neuroscience.2012.07.047 22863672

[B50] Kohda K. Harada K. Kato K. Hoshino A. Motohashi J. Yamaji T. (2007). Glucocorticoid receptor activation is involved in producing abnormal phenotypes of single-prolonged stress rats: a putative post-traumatic stress disorder model. *Neuroscience* 148 22–33. 10.1016/j.neuroscience.2007.05.041 17644267

[B51] Koob G. F. Volkow N. D. (2010). Neurocircuitry of addiction. *Neuropsychopharmacology* 35 217–238. 10.1038/npp.2009.110 19710631 PMC2805560

[B52] Krugers H. J. Hoogenraad C. C. Groc L. (2010). Stress hormones and AMPA receptor trafficking in synaptic plasticity and memory. *Nat. Rev. Neurosci.* 11 675–681. 10.1038/nrn2913 20820185

[B53] Lee C. C. Huang C. C. Hsu K. S. (2015). The phospholipid-binding protein SESTD1 negatively regulates dendritic spine density by interfering with Rac1-Trio8 signaling pathway. *Sci. Rep.* 5:13250. 10.1038/srep13250 26272757 PMC4536496

[B54] Leuner B. Shors T. J. (2013). Stress, anxiety, and dendritic spines: what are the connections? *Neuroscience* 251 108–119. 10.1016/j.neuroscience.2012.04.021 22522470

[B55] Liberzon I. Krstov M. Young E. A. (1997). Stress-restress: effects on ACTH and fast feedback. *Psychoneuroendocrinology* 22 443–453. 10.1016/S0306-4530(97)00044-9 9364622

[B56] Lin C.-C. Tung C.-S. Liu Y.-P. (2016). Escitalopram reversed the traumatic stress-induced depressed and anxiety-like symptoms but not the deficits of fear memory. *Psychopharmacology* 233 1135–1146. 10.1007/s00213-015-4194-5 26740318

[B57] Lippi G. Fernandes C. C. Ewell L. A. John D. Romoli B. Curia G. (2016). MicroRNA-101 regulates multiple developmental programs to constrain excitation in adult neural networks. *Neuron* 92 1337–1351. 10.1016/j.neuron.2016.11.017 27939580 PMC5182124

[B58] Lu V. B. Ballanyi K. Colmers W. F. Smith P. A. (2007). Neuron type-specific effects of brain-derived neurotrophic factor in rat superficial dorsal horn and their relevance to ‘central sensitization’. *J. Physiol.* 584 (Pt 2), 543–563. 10.1113/jphysiol.2007.141267 17761774 PMC2277149

[B59] Maletic-Savatic M. Malinow R. Svoboda K. (1999). Rapid dendritic morphogenesis in CA1 hippocampal dendrites induced by synaptic activity. *Science* 283 1923–1927. 10.1126/science.283.5409.1923 10082466

[B60] Malgaroli A. Tsien R. W. (1992). Glutamate-induced long-term potentiation of the frequency of miniature synaptic currents in cultured hippocampal neurons. *Nature* 357 134–139. 10.1038/357134a0 1349728

[B61] Maroun M. Ioannides P. J. Bergman K. L. Kavushansky A. Holmes A. Wellman C. L. (2013). Fear extinction deficits following acute stress associate with increased spine density and dendritic retraction in basolateral amygdala neurons. *Eur. J. Neurosci.* 38 2611–2620. 10.1111/ejn.12259 23714419 PMC3773716

[B62] McDonald A. J. (1982). Neurons of the lateral and basolateral amygdaloid nuclei: a Golgi study in the rat. *J. Comp. Neurol.* 212 293–312. 10.1002/cne.902120307 6185547

[B63] Mellman T. A. Alim T. Brown D. D. Gorodetsky E. Buzas B. Lawson W. B. (2009). Serotonin polymorphisms and posttraumatic stress disorder in a trauma exposed African American population. *Depress. Anxiety* 26 993–997. 10.1002/da.20627 19842167 PMC2963151

[B64] Mitra R. Jadhav S. McEwen B. S. Vyas A. Chattarji S. (2005). Stress duration modulates the spatiotemporal patterns of spine formation in the basolateral amygdala. *Proc. Natl. Acad. Sci. U.S.A.* 102 9371–9376. 10.1073/pnas.0504011102 15967994 PMC1166638

[B65] Miura Y. Naka M. Matsuki N. Nomura H. (2012). Differential calcium dependence in basal and forskolin-potentiated spontaneous transmitter release in basolateral amygdala neurons. *Neurosci. Lett.* 529 1–6. 10.1016/j.neulet.2012.09.015 22989859

[B66] Mizunuma M. Norimoto H. Tao K. Egawa T. Hanaoka K. Sakaguchi T. (2014). Unbalanced excitability underlies offline reactivation of behaviorally activated neurons. *Nat. Neurosci.* 17 503–505. 10.1038/nn.3674 24633127

[B67] Moench K. M. Wellman C. L. (2015). Stress-induced alterations in prefrontal dendritic spines: implications for post-traumatic stress disorder. *Neurosci. Lett.* 601 41–45. 10.1016/j.neulet.2014.12.035 25529195

[B68] Montalbano A. Baj G. Papadia D. Tongiorgi E. Sciancalepore M. (2013). Blockade of BDNF signaling turns chemically-induced long-term potentiation into long-term depression. *Hippocampus* 23 879–889. 10.1002/hipo.22144 23674394

[B69] Nagode D. A. Meng X. Winkowski D. E. Smith E. Khan-Tareen H. Kareddy V. (2017). Abnormal development of the earliest cortical circuits in a mouse model of autism spectrum disorder. *Cell Rep.* 18 1100–1108. 10.1016/j.celrep.2017.01.006 28147267 PMC5488290

[B70] Noguchi J. Matsuzaki M. Ellis-Davies G. C. Kasai H. (2005). Spine-neck geometry determines NMDA receptor-dependent Ca2+ signaling in dendrites. *Neuron* 46 609–622. 10.1016/j.neuron.2005.03.015 15944129 PMC4151245

[B71] Oe Y. Tominaga-Yoshino K. Hasegawa S. Ogura A. (2013). Dendritic spine dynamics in synaptogenesis after repeated LTP inductions: dependence on pre-existing spine density. *Sci. Rep.* 3:1957. 10.1038/srep01957 23739837 PMC3674431

[B72] Oh J. Y. Kim Y. K. Kim S. N. Lee B. Jang J. H. Kwon S. (2018). Acupuncture modulates stress response by the mTOR signaling pathway in a rat post-traumatic stress disorder model. *Sci. Rep.* 8:11864. 10.1038/s41598-018-30337-5 30089868 PMC6082850

[B73] Olaya B. Alonso J. Atwoli L. Kessler R. C. Vilagut G. Haro J. M. (2015). Association between traumatic events and post-traumatic stress disorder: results from the ESEMeD-Spain study. *Epidemiol. Psychiatr. Sci.* 24 172–183. 10.1017/S2045796014000092 24565167 PMC4143480

[B74] Padival M. A. Blume S. R. Rosenkranz J. A. (2013). Repeated restraint stress exerts different impact on structure of neurons in the lateral and basal nuclei of the amygdala. *Neuroscience* 246 230–242. 10.1016/j.neuroscience.2013.04.061 23660193 PMC3722557

[B75] Papoutsi A. Kastellakis G. Psarrou M. Anastasakis S. Poirazi P. (2014). Coding and decoding with dendrites. *J. Physiol. Paris* 108 18–27. 10.1016/j.jphysparis.2013.05.003 23727338

[B76] Pitman R. K. Rasmusson A. M. Koenen K. C. Shin L. M. Orr S. P. Gilbertson M. W. (2012). Biological studies of post-traumatic stress disorder. *Nat. Rev. Neurosci.* 13 769–787. 10.1038/nrn3339 23047775 PMC4951157

[B77] Pratchett L. C. Daly K. Bierer L. M. Yehuda R. (2011). New approaches to combining pharmacotherapy and psychotherapy for posttraumatic stress disorder. *Expert Opin. Pharmacother.* 12 2339–2354. 10.1517/14656566.2011.604030 21819273

[B78] Qiao H. Li M. X. Xu C. Chen H. B. An S. C. Ma X. M. (2016). Dendritic spines in depression: what we learned from animal models. *Neural Plast.* 2016:8056370. 10.1155/2016/8056370 26881133 PMC4736982

[B79] Rainnie D. G. Bergeron R. Sajdyk T. J. Patil M. Gehlert D. R. Shekhar A. (2004). Corticotrophin releasing factor-induced synaptic plasticity in the amygdala translates stress into emotional disorders. *J. Neurosci.* 24 3471–3479. 10.1523/JNEUROSCI.5740-03.2004 15071094 PMC6729749

[B80] Rao R. P. Anilkumar S. McEwen B. S. Chattarji S. (2012). Glucocorticoids protect against the delayed behavioral and cellular effects of acute stress on the amygdala. *Biol. Psychiatry* 72 466–475. 10.1016/j.biopsych.2012.04.008 22572034 PMC3753225

[B81] Rauch S. L. Whalen P. J. Shin L. M. McInerney S. C. Macklin M. L. Lasko N. B. (2000). Exaggerated amygdala response to masked facial stimuli in posttraumatic stress disorder: a functional MRI study. *Biol. Psychiatry* 47 769–776. 10.1016/S0006-3223(00)00828-3 10812035

[B82] Rochefort N. L. Konnerth A. (2012). Dendritic spines: from structure to in vivo function. *EMBO Rep.* 13 699–708. 10.1038/embor.2012.102 22791026 PMC3410382

[B83] Ronzoni G. Del Arco A. Mora F. Segovia G. (2016). Enhanced noradrenergic activity in the amygdala contributes to hyperarousal in an animal model of PTSD. *Psychoneuroendocrinology* 70 1–9. 10.1016/j.psyneuen.2016.04.018 27131036

[B84] Rosenkranz J. A. Venheim E. R. Padival M. (2010). Chronic stress causes amygdala hyperexcitability in rodents. *Biol. Psychiatry* 67 1128–1136. 10.1016/j.biopsych.2010.02.008 20378100 PMC2882519

[B85] Ryan S. J. Ehrlich D. E. Jasnow A. M. Daftary S. Madsen T. E. Rainnie D. G. (2012). Spike-timing precision and neuronal synchrony are enhanced by an interaction between synaptic inhibition and membrane oscillations in the amygdala. *PLoS One* 7:e35320. 10.1371/journal.pone.0035320 22563382 PMC3338510

[B86] Saghir Z. Syeda J. N. Muhammad A. S. Balla Abdalla T. H. (2018). The Amygdala, sleep debt, sleep deprivation, and the emotion of anger: a possible connection? *Cureus* 10:e2912. 10.7759/cureus.2912 30186717 PMC6122651

[B87] Sastry B. R. Bhagavatula L. S. (1996). Quantal release of transmitter at a central synapse. *Neuroscience* 75 987–992. 10.1016/0306-4522(96)00348-X 8938734

[B88] Schilling S. Mehr A. Ludewig S. Stephan J. Zimmermann M. August A. (2017). APLP1 is a synaptic cell adhesion molecule, supporting maintenance of dendritic spines and basal synaptic transmission. *J. Neurosci.* 37 5345–5365. 10.1523/JNEUROSCI.1875-16.2017 28450540 PMC6596463

[B89] Scott K. M. Koenen K. C. Aguilar-Gaxiola S. Alonso J. Angermeyer M. C. Benjet C. (2013). Associations between lifetime traumatic events and subsequent chronic physical conditions: a cross-national, cross-sectional study. *PLoS One* 8:e80573. 10.1371/journal.pone.0080573 24348911 PMC3864645

[B90] Seal K. H. Metzler T. J. Gima K. S. Bertenthal D. Maguen S. Marmar C. R. (2009). Trends and risk factors for mental health diagnoses among Iraq and Afghanistan veterans using department of veterans affairs health care, 2002-2008. *Am. J. Public Health* 99 1651–1658. 10.2105/AJPH.2008.150284 19608954 PMC2724454

[B91] Segal M. (2010). Dendritic spines, synaptic plasticity and neuronal survival: activity shapes dendritic spines to enhance neuronal viability. *Eur. J. Neurosci.* 31 2178–2184. 10.1111/j.1460-9568.2010.07270.x 20550565

[B92] Shekhar A. Truitt W. Rainnie D. Sajdyk T. (2005). Role of stress, corticotrophin releasing factor (CRF) and amygdala plasticity in chronic anxiety. *Stress* 8 209–219. 10.1080/10253890500504557 16423710

[B93] Simkus C. R. Stricker C. (2002). The contribution of intracellular calcium stores to mEPSCs recorded in layer II neurones of rat barrel cortex. *J. Physiol.* 545 521–535. 10.1113/jphysiol.2002.022103 12456831 PMC2290677

[B94] Souza R. R. Noble L. J. McIntyre C. K. (2017). Using the Single Prolonged Stress Model to Examine the Pathophysiology of PTSD. *Front. Pharmacol.* 8:615. 10.3389/fphar.2017.00615 28955225 PMC5600994

[B95] Spruston N. (2008). Pyramidal neurons: dendritic structure and synaptic integration. *Nat. Rev. Neurosci.* 9 206–221. 10.1038/nrn2286 18270515

[B96] Sun X. D. Chen W. B. Sun D. Huang J. Li Y. Q. Pan J. X. (2018). Neogenin in Amygdala for neuronal activity and information processing. *J. Neurosci.* 38 9600–9613. 10.1523/JNEUROSCI.0433-18.2018 30228230 PMC6209834

[B97] Suo L. Zhao L. Si J. Liu J. Zhu W. Chai B. (2013). Predictable chronic mild stress in adolescence increases resilience in adulthood. *Neuropsychopharmacology* 38 1387–1400. 10.1038/npp.2013.67 23478858 PMC3682155

[B98] Suvrathan A. Bennur S. Ghosh S. Tomar A. Anilkumar S. Chattarji S. (2014). Stress enhances fear by forming new synapses with greater capacity for long-term potentiation in the amygdala. *Philos. Trans. R. Soc. Lond. B Biol. Sci.* 369:20130151. 10.1098/rstb.2013.0151 24298153 PMC3843883

[B99] Takahashi T. Morinobu S. Iwamoto Y. Yamawaki S. (2006). Effect of paroxetine on enhanced contextual fear induced by single prolonged stress in rats. *Psychopharmacology* 189 165–173. 10.1007/s00213-006-0545-6 17031709

[B100] Truitt W. A. Sajdyk T. J. Dietrich A. D. Oberlin B. McDougle C. J. Shekhar A. (2007). From anxiety to autism: spectrum of abnormal social behaviors modeled by progressive disruption of inhibitory neuronal function in the basolateral amygdala in Wistar rats. *Psychopharmacology* 191 107–118. 10.1007/s00213-006-0674-y 17277936

[B101] Udagawa T. Fujioka Y. Tanaka M. Honda D. Yokoi S. Riku Y. (2015). FUS regulates AMPA receptor function and FTLD/ALS-associated behaviour via GluA1 mRNA stabilization. *Nat. Commun.* 6:7098. 10.1038/ncomms8098 25968143 PMC4479014

[B102] Ueno T. Yamada J. Nishijima H. Arai A. Migita K. Baba M. (2014). Morphological and electrophysiological changes in intratelencephalic-type pyramidal neurons in the motor cortex of a rat model of levodopa-induced dyskinesia. *Neurobiol. Dis.* 64 142–149. 10.1016/j.nbd.2013.12.014 24398173

[B103] Vyas A. Jadhav S. Chattarji S. (2006). Prolonged behavioral stress enhances synaptic connectivity in the basolateral amygdala. *Neuroscience* 143 387–393. 10.1016/j.neuroscience.2006.08.003 16962717

[B104] Vyas A. Mitra R. Shankaranarayana Rao B. S. Chattarji S. (2002). Chronic stress induces contrasting patterns of dendritic remodeling in hippocampal and amygdaloid neurons. *J. Neurosci.* 22 6810–6818. 10.1523/JNEUROSCI.22-15-06810.2002 12151561 PMC6758130

[B105] Wang X. X. Li J. T. Xie X. M. Gu Y. Si T. M. Schmidt M. V. (2017). Nectin-3 modulates the structural plasticity of dentate granule cells and long-term memory. *Transl. Psychiatry* 7:e1228. 10.1038/tp.2017.196 28872640 PMC5639241

[B106] Wen L. Han F. Shi Y. Li X. (2016). Role of the endoplasmic reticulum pathway in the medial prefrontal cortex in post-traumatic stress disorder model rats. *J. Mol. Neurosci.* 59 471–482. 10.1007/s12031-016-0755-2 27112439

[B107] Wissman A. M. McCollum A. F. Huang G. Z. Nikrodhanond A. A. Woolley C. S. (2011). Sex differences and effects of cocaine on excitatory synapses in the nucleus accumbens. *Neuropharmacology* 61 217–227. 10.1016/j.neuropharm.2011.04.002 21510962 PMC3105198

[B108] Xue Y. X. Zhu Z. Z. Han H. B. Liu J. F. Meng S. Q. Chen C. (2015). Overexpression of protein kinase mzeta in the prelimbic cortex enhances the formation of long-term fear memory. *Neuropsychopharmacology* 40 2146–2156. 10.1038/npp.2015.56 25722116 PMC4613603

[B109] Yamamoto S. Morinobu S. Fuchikami M. Kurata A. Kozuru T. Yamawaki S. (2008). Effects of single prolonged stress and D-cycloserine on contextual fear extinction and hippocampal NMDA receptor expression in a rat model of PTSD. *Neuropsychopharmacology* 33 2108–2116. 10.1038/sj.npp.1301605 17957211

[B110] Yang G. Pan F. Gan W. B. (2009). Stably maintained dendritic spines are associated with lifelong memories. *Nature* 462 920–924. 10.1038/nature08577 19946265 PMC4724802

[B111] Yang X. D. Liao X. M. Uribe-Marino A. Liu R. Xie X. M. Jia J. (2015). Stress during a critical postnatal period induces region-specific structural abnormalities and dysfunction of the prefrontal cortex via CRF1. *Neuropsychopharmacology* 40 1203–1215. 10.1038/npp.2014.304 25403725 PMC4367464

[B112] Yasmin F. Saxena K. McEwen B. S. Chattarji S. (2016). The delayed strengthening of synaptic connectivity in the amygdala depends on NMDA receptor activation during acute stress. *Physiol. Rep.* 4:e13002. 10.14814/phy2.13002 27798355 PMC5099964

[B113] Yehuda R. Southwick S. M. Krystal J. H. Bremner D. Charney D. S. Mason J. W. (1993). Enhanced suppression of cortisol following dexamethasone administration in posttraumatic stress disorder. *Am. J. Psychiatry* 150 83–86. 10.1176/ajp.150.1.83 8417586

[B114] Yin H. H. Mulcare S. P. Hilario M. R. Clouse E. Holloway T. Davis M. I. (2009). Dynamic reorganization of striatal circuits during the acquisition and consolidation of a skill. *Nat. Neurosci.* 12 333–341. 10.1038/nn.2261 19198605 PMC2774785

[B115] Zhang J. Y. Liu T. H. He Y. Pan H. Q. Zhang W. H. Yin X. P. (2019). Chronic stress remodels synapses in an Amygdala circuit-specific manner. *Biol. Psychiatry* 85 189–201. 10.1016/j.biopsych.2018.06.019 30060908 PMC6747699

[B116] Zhang Y. Gandhi P. R. Standifer K. M. (2012). Increased nociceptive sensitivity and nociceptin/orphanin FQ levels in a rat model of PTSD. *Mol. Pain* 8:76. 10.1186/1744-8069-8-76 23082795 PMC3543245

[B117] Zoladz P. R. Diamond D. M. (2013). Current status on behavioral and biological markers of PTSD: a search for clarity in a conflicting literature. *Neurosci. Biobehav. Rev.* 37 860–895. 10.1016/j.neubiorev.2013.03.024 23567521

