# Corrigendum: Traumatic stress produces delayed alterations of synaptic plasticity in basolateral amygdala

**DOI:** 10.3389/fpsyg.2024.1486849

**Published:** 2024-09-26

**Authors:** Huan-Huan Zhang, Shi-Qiu Meng, Xin-Yi Guo, Jing-Liang Zhang, Wen Zhang, Ya-Yun Chen, Lin Lu, Jian-Li Yang, Yan-Xue Xue

**Affiliations:** ^1^Department of Psychiatry, Tianjin Medical University, Tianjin, China; ^2^Department of Clinical Psychology, Tianjin Medical University General Hospital, Tianjin, China; ^3^National Institute on Drug Dependence, Peking University, Beijing, China; ^4^State Key Laboratory of Natural and Biomimetic Drugs, Department of Molecular and Cellular Pharmacology, Peking University School of Pharmaceutical Sciences, Beijing, China; ^5^Department of Medicinal Chemistry and Molecular Pharmacology, Purdue University College of Pharmacy and Purdue Institute for Integrative Neuroscience, West Lafayette, IN, United States; ^6^Peking University Sixth Hospital/Peking University Institute of Mental Health, Peking University, Beijing, China

**Keywords:** single prolonged stress, post-traumatic stress disorder, dendritic spines, synaptic plasticity, basolateral amygdala

In the published article, there was an error in the legend for Figure 2A as published. The low-power image of dendritic spines of BLA was mistakenly labeled as from the control rats. The corrected Figure 2 caption appears below.

Figure 2. Effect of SPS paradigms on spine density of BLA pyramidal neurons. **(A)** Low-power image of dendritic spines of BLA from SPS-treated rats. Scale bar = 10 μm. Dendritic spines were classified based on morphology: thin dendritic spines have thin head and long neck (indicated by green arrows), mushroom dendritic spines come with large head and short neck (indicated by yellow arrows) and stubby dendritic spines have large head but no apparent neck (indicated by red arrows). Scale bar = 10 μm. **(B)** High-power image of representative dendrite segments (scale bar = 10 μm). **(C)** Spine density in BLA pyramidal dendrite segments in different experimental conditions (animals, rats = 5; segments, *n* = 5–8, total dendritic length = 40–70 μm). **(D–F)** Average density in mushroom **(D)**, thin **(E)**, and stubby **(F)** spines in BLA pyramidal dendrite segments sampled from four groups: NO SPS(1d)/SPS(1d)/NO SPS(10d)/SPS(10d). ^#^Different from SPS(1d) group, ^#*^Different from NO SPS group at each post-SPS day, ^#*^p < 0.05, two-way ANOVA. Data are shown as means ± SEM.

The authors apologize for this error and state that this does not change the scientific conclusions of the article in any way. The original article has been updated.

