# Supplementary figures and images for: Traumatic Stress Produces Delayed Alterations of Synaptic Plasticity in Basolateral Amygdala
Source: Front Psychol. 2019 Oct 25;10:2394. doi: 10.3389/fpsyg.2019.02394 (PMC6824323; doi:10.3389/fpsyg.2019.02394)

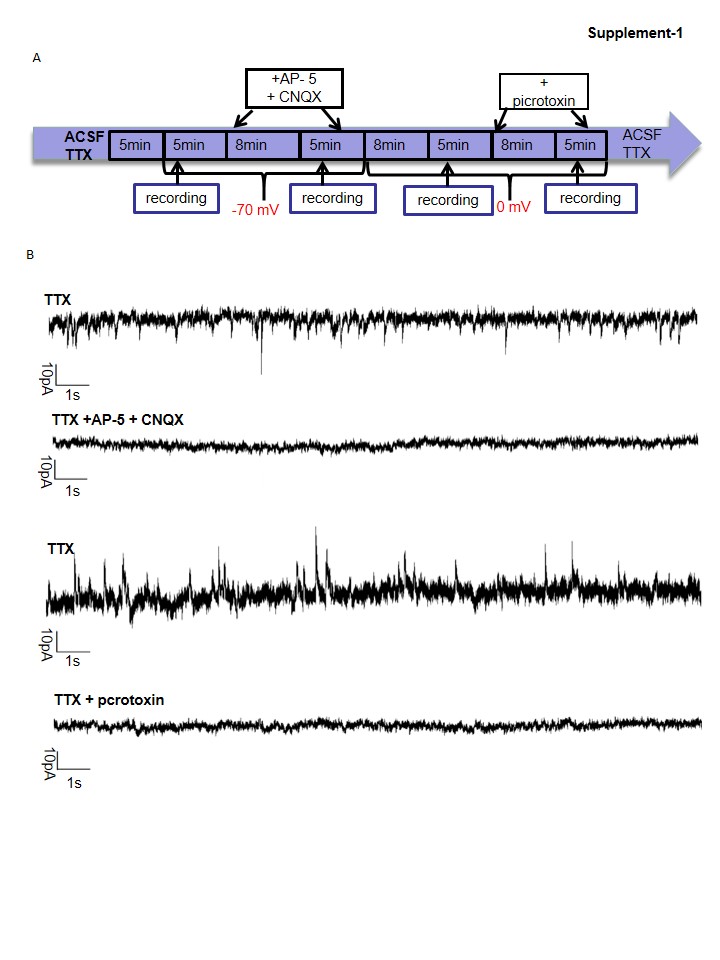

Supplement: FIGURE S1 — (A) Experimental timeline. (B) In defect of antagonists of glutamatergic or GABAergic receptors, mEPSCs and mIPSCs were recorded at clamped voltages of −70 and 0 mV. After adding 20 μM CNQX and 50 μM AP5, the postsynaptic currents recorded at −70 mV were blocked, while those recorded at 0 mV were suppressed by 50 μM picrotoxin. [file Image_1.JPEG]

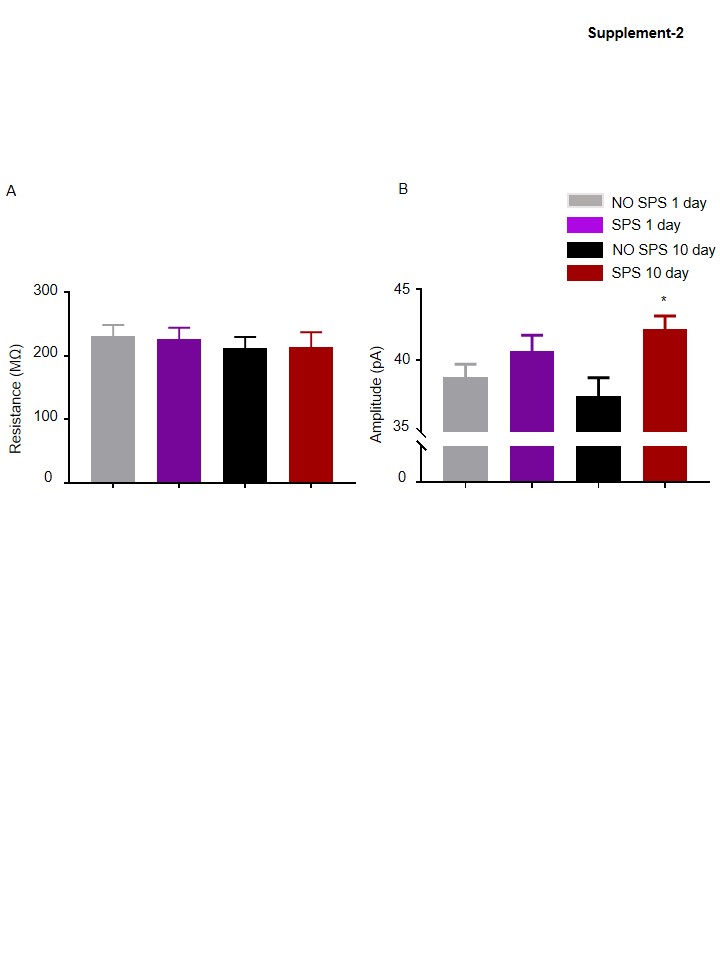

Supplement: FIGURE S2 — (A) The input resistance of BLA pyramidal neurons among the different experimental conditions. (B) Large amplitude events (>30 pA) of mEPSCs in BLA pyramidal neurons from four groups: NO SPS(1d)/SPS(1d)/NO SPS(10d)/SPS(10d). ∗Different from NO SPS groups. One-way ANOVA, ∗p < 0.05. [file Image_2.JPEG]
